# French Version of the Antiphasic Digits-in-Noise Test for Smartphone Hearing Screening

**DOI:** 10.3389/fpubh.2021.725080

**Published:** 2021-10-14

**Authors:** Jean-Charles Ceccato, Marie-Josée Duran, De Wet Swanepoel, Cas Smits, Karina C. De Sousa, Lewis Gledhill, Frédéric Venail, Jean-Luc Puel

**Affiliations:** ^1^INM, Univ Montpellier, Inserm, Montpellier, France; ^2^Audiocampus, UFR Pharmacie, Univ Montpellier, Montpellier, France; ^3^Fondation Pour l'Audition, Paris, France; ^4^Department of Speech-Language Pathology and Audiology, Univ Pretoria, Pretoria, South Africa; ^5^Department of Otolaryngology-Head and Neck Surgery, Amsterdam UMC, Amsterdam Public Health Research Institute, Vrije Universiteit Amsterdam Ear & Hearing, Amsterdam, Netherlands; ^6^Otology and Neurotology Unit, Univ Montpellier, CHU Montpellier, Montpellier, France

**Keywords:** hearing loss, French antiphasic DIN test, smartphone, Höra app, speech-in-noise (SIN)

## Abstract

In France 58% of persons with hearing loss still do not wear hearing aids. Pure-tone audiometry is the traditional gold standard in assessment and screening of hearing impairment, but it requires the use of calibrated devices and soundproof booth. The antiphasic digits-in-noise (DIN) test does not require calibrated material and can run on a standard headset or earbuds connected to a smartphone or a computer. The DIN test is highly correlated with pure tone audiometry and has already shown to be effective in hearing loss screening in its English version promoted by the WHO. The aim of the present study was to develop and validate a French version of the antiphasic DIN test for implementation on a national screening test offered as a smartphone app. The audio files recorded from a French native female speaker were selected and normalized in intensity according to their recognition probability. The French DIN test application was then tested on normal hearing- and hearing-impaired subjects. Based on the strong correlation between pure tone audiometry (PTA) and DIN SRT, we calculated ROC curves and Z-score. For PTA > 20 dB HL, a SNR cutoff of 12.9 dB corresponds to a sensitivity and specificity of 0.96 and 0.93, respectively. To detect moderate and more severe hearing loss (PTA > 40 dB HL), the SNR cutoff was −10.9 dB, corresponding to a sensitivity and specificity of 0.99 and 0.83, respectively. The Z-score was calculated to define statistical criteria of normality for speech-in-noise evaluation. While a score of 0 roughly corresponds to the normality (DIN SRT = −15.4 dB SNR), a subject with DIN SRT > −12.2 (Z-score > 2) is ranked in the hearing loss population. Next, the French antiphasic DIN test was implemented in the Höra iOS and Android apps. In total, 19,545 Höra tests were completed and analyzed. Three quarters of them were classified as normal (74 %) and one quarter presented mild (9%) or more severe loss (17%). Together, results argue for the use of the French version of antiphasic DIN test in the general population to improve the screening of hearing-impaired individuals.

## Introduction

Hearing loss burden on health and quality of life is often underestimated by public authorities, health care professionals and the public. In the world, hearing loss affects around 20% (1.5 billion people) of the population with an estimated 5.5% (430 million) experiencing significant hearing loss. In France as in other high-income countries, the prevalence is expected to increase rapidly over the next three decades due to an aging population and noise exposure of younger people (1).

The impact of untreated hearing loss is far reaching with economic cost estimates of ~22.5 billion Euros in France, and 225 billion in Europe (1). On an individual level, untreated hearing loss is associated with social isolation, increased risk of depression, cognitive decline, dementia, and hospitalization (2). On the other hand, hearing aid use is associated with significant improvement in social, psychologic, emotional, and physical aspects of the lives of persons with hearing loss with all degrees of hearing loss (3–5). Hearing loss has recently been identified as the most significant modifiable risk factor in mid-life for dementia (6), which emphasizes the importance of early detection and timely intervention.

In France 58% of persons with hearing loss still do not wear hearing aids (7). For one third of them, the cause relies on the lack of screening since most of cases are referred after self-declaration to general practitioners or targeted hearing loss screening campaigns. For the remainder, either no medical recommendation has been made or the cost of hearing aids has been a barrier (3, 7). Although French Health Insurance recently adopted regulation for full reimbursement of hearing aids (8), the absence of a national screening strategy for hearing loss limits widespread uptake.

Pure-tone audiometry is the traditional gold standard in assessment and screening of hearing. However, it includes inherent limitations that make large-scale population-based screening programs difficult. Firstly, pure tone audiometry requires the use of calibrated devices that are typically set in a sound treated environments and require trained professionals to operate. Secondly, it is insensitive to the early and specific patient complaints such as difficulty with speech understanding in noise (9). Although some efforts in the development of self-testing applications for pure tone hearing thresholds on laptops or mobile devices have been attempted, accurate testing is difficult due to the challenge of uncalibrated testing across various digital devices and headphones (10–17). An alternative to pure tone audiometry is the use of speech-in-noise testing as a screening tool. The suprathreshold and relative measure of a signal-to-noise ratio (SNR) does not require absolute calibrated levels of noise or speech. As a result hearing screening is made possible using non-calibrated devices such as smartphones coupled with generic headphones (18).

Pioneering experiments from Wilson and Smits teams (19–23) investigated the possibility to use digit pairs or digit triplets for speech-in-noise testing. First developed to screen the Dutch population using landline phone, digits and noise were presented in one ear (monaurally) (22, 23). The digits-in-noise test (DIN test, or “digit-triplet test”) was then adapted in different languages [e.g., Dutch, German, British, Australian, Polish, Swiss, and French, see Van de Borre et al. for review (24)]. Different platforms have been used for hearing screening (telephone, internet, tablets, smartphones) and different populations have been targeted, mainly adults, but also young school children (25–28). The presentation method of the stimuli differs as well. In most of the studies, both ears are tested separately. In others, stimuli are presented binaurally, for kids for example (18, 28–32). Presenting speech materials binaurally involves more central auditory processes than monaural presentation (33) and halves test duration which reduces task dropout compared to sequentially testing each ear. Since the diotic test strongly relies on the better ear, asymmetric, or unilateral hearing losses are easily missed (31, 34). Moreover, both monaural and binaural/diotic speech-in-noise tests are insensitive to conductive hearing loss (CHL) (31). Recently, the use of antiphasic stimuli has been explored and standardized (31). Presenting stimuli in opposite phase between ears with masking noise interaurally in-phase enable binaural masking release that improve stimuli perception (35, 36). This phenomenon, called binaural masking level difference is frequency dependant and relies on well-preserved and symmetric hearing to be effective. Compared to binaural presentation, speech reception thresholds (SRT) of the antiphasic DIN test are more strongly correlated with the worse ear pure tone average (PTA) across 0.5, 1, 2, and kHz than with the better ear PTA. The SRT distribution range is also wider for the antiphasic DIN SRTs than for diotic DIN SRTs, and differences in SRT are larger between normal hearing and hearing loss persons (31). As a result, antiphasic digits presentation markedly improved the specificity (0.8 vs. 0.71) and the sensitivity (0.9 vs. 0.75) to symmetric and asymmetric sensorineural hearing loss as well as conductive hearing loss compared to diotic presentation (31, 36, 37).

Combined with smartphone technology the antiphasic DIN screening is accessible to a large global audience. For example, the HearZA app was used successfully in a national hearing screening campaign in South Africa, with a binaural test. The World Health Organization (WHO) has adopted this antiphasic DIN screening approach in the use of their hearWHO smartphone application available in English, Spanish and Mandarin (38). To date no antiphasic DIN test has yet been developed in French for a digital platform like smartphone. The purpose of the present study was to develop and to validate a French version of the antiphasic DIN for a smartphone app.

## Materials and Methods

According to Jardé's law regulating biomedical research in France, this type of evaluation is considered as non-interventional research and did not require institutional review board approval. All eligible participants were informed of the study aims and procedures and provided consent before participation.

### Digits Recording and Level Normalization

#### Recording and Processing the Speech Material

**Figure 1:**
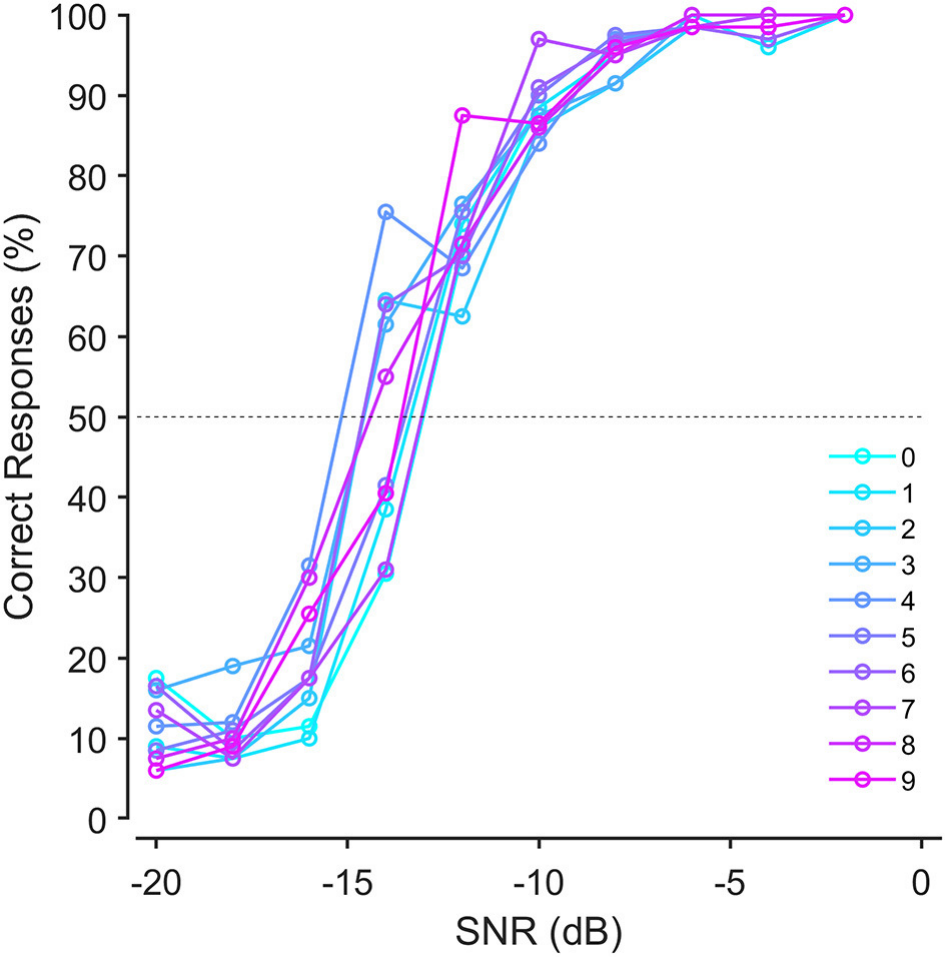
Average speech recognition probabilities for single digits-in-noise before equalization.

French mono- and bi-syllabic digits (0–9) were selected as speech material. Single-digit recordings were made from a native French female speaker in a sound-proof booth. A carrier sentence “le chiffre” was uttered before pronouncing each digit to allow natural intonation. A microphone (Blue Yeti Microphone) was held ~5 cm from the speakers' mouth during recordings. The speaker was asked to read four lists of randomly ordered digits. Thus, each digit appeared four times. The recordings were digitalized at 44,1 kHz with floating 32 bits resolution using Audacity software (Audacity). Each digit was then cropped manually from the list and stored separately in WAV files using Audacity software. One speech-language therapist and one phonetic academic teacher rated the four recordings of each digit, according to the naturalness, articulation, voice quality, intonation, and speed of production. The final list of digits was compiled using the best rated digits for digits 0–9. The masking noise was generated by shaping a white noise (using FIR filter in Matlab) with the long-term average spectrum of the 10 selected digits (Figure 1). The recording level (RMS in Volt) of the masking noise was set to the average recording level of the 10 digits without any silences (18, 39).

#### Equalization

Digits were equalized according to their recognition probability. Equalizing digits by applying level corrections to the digits ensured that each digit had a 50% chance of being recognized correctly at the same SNR. To do so, we recruited 20 normal-hearing (both ears) participants aged 20–33 years, with pure-tone thresholds <20 dB HL from 250 to 8,000 Hz. A custom Matlab script was used to generate the sequences of digits superimposed with noise on a laptop (MacBook Pro) that were presented monaurally through circumaural headphones (Sony WH1000XM3). Four lists of 10 digits were successively presented at 10 different SNRs decreasing from −2 to −20 dB in 2 dB steps. For each SNR level the 10 digits were presented randomly. The noise started 500 ms before and finished 500 ms after each digit. The participant was forced to choose a digit, even if it was not recognized (forced-choice procedure). The psychometric curves of recognition for each digit were fitted with a logistic function to determine the speech reception threshold (SRT, i.e., the SNR corresponding to a 50% recognition probability (Figure 1). Each digit's recording level was then adjusted using the difference between the SRT of each digit and the average SRT of all the digits (±0.4 dB maximum).

### Validation of the French Antiphasic DIN Test

Before administering the DIN test, pure-tone audiometry was completed for all the participants. The two ears were evaluated with air and bone conduction audiometry across the frequencies of 500, 1,000, 2,000, and 4,000 Hz.

#### Study Design

Pure tone audiometry was performed in a sound booth with a digital audiometer (Audyx) equipped with a supra-aural TDH39 headphone for air conduction and a bone vibrator B71 for bone conduction. The hearing status was determined according to the pure tone threshold average at 500, 1,000, 2,000, and 4,000 Hz. The normal-hearing participants with PTA ≤ 20 dBHL in both ears were students at the University of Montpellier and of the Institute for Neuroscience of Montpellier, relatives of the authors or accompanying hearing loss people. The participants with hearing loss came from the University Hospital of Montpellier or private clinics.

#### Population of Reference

The beta version of the French antiphasic DIN test was evaluated in a normative reference population, including normal and hearing loss subjects of different ages (*n* = 167). The participants (77 women and 90 men) were aged from 19 to 90 years (mean age 56 years of age ±22). The hearing status of the subject was classified according to the recommendation of the International Bureau for Audiophonology (BIAP) (40). Among 167 subjects tested, 66 had normal-hearing (PTA ≤ 20 dB HL, 32.5 years of age ±11.5), 75 symmetric sensorineural hearing loss (PTA > 20 dBHL, 71.28 years of age ±10.6), 19 unilateral or asymmetric hearing loss (PTA difference between both ears >10 dB, 72.7 years of age ±10). In addition, 7 mixed hearing loss based on air bone gap criteria (PTA difference between bone and air conduction > 20 dB and bone conduction PTA > 20 dB, 74.8 years of age ±8.5) and other test results such as tympanometry and otoscopy.

#### DIN Test Procedure and Equipment

DIN test was developed in a webapp working on Google Chrome running on a laptop connected to a commercial headphone (Sony WH 1000 XM3). The selected digits were organized in 120 triplets stored in stereo files. The participants were informed on how to enter the digit responses on the computer keyboard. Before starting the test, they were asked to adjust the loudness of the digit-triplets to a comfortable listening level. The noise was presented in-phase (diotic) in both ears, and the digits were antiphasic between the two ears. The noise was present during the entire digit triplet sequence and started 500 ms before the first digit. Successive digits within a triplet were separated by 200 ms intervals. The initial SNR was fixed at 0 dB and the SNR change was obtained by varying the noise level when the SNR is positive, or by varying the digits level when the SNR is negative (31). To prevent possible learning of the masking noise (41), noise refreshment was ensured for each trial by creating a long noise file where different fragments were randomly selected. For each presentation, the application randomly selected 3 different digits to produce the presented triplet superimposed with noise. The subject was required to enter the digits they recognized (or guessed) directly on the laptop as they would perform on the smartphone application. Depending on the answers, the signal-to-noise ratio was adjusted following a 1-up, 1-down adaptive procedure using step size of 4 dB SNR for the first 3 steps, thereafter continuing in 2 dB steps. After 23 triplets presentations, the test stops and the DIN SRT was calculated as the average of the last 19 SNRs (18, 22). Subjects were tested only once without training to obtain scores that reflects more closely the results that could be obtained on the smartphone application for naïve listeners. Adding a training phase in the smartphone application may be counterproductive because the increased test duration will reduce uptake and completion.

The French smartphone-based antiphasic digits-in-noise hearing application (Höra) was developed on iOS and Android and is available for free in the Apple store and Google Play Store. The mobile app can be used with standard headphones or earphones. When the application is launched, a tutorial screen appears to inform the subject how to use the application. The subject is instructed to enter a name or nickname (use is fully anonymous), gender and birthdate. Next, the subject is invited to put on the smartphone headset and adjust the intensity of the continuously presented digit-triplets to a comfortable listening level using a scroll bar. When ready, a “Start Test” button allows the subject to begin testing. A pop-up number pad appears after each presentation of the digits to allow the subject to enter his/her responses. A personalized score out of 100 was deduced from the range of SNRs of our reference population. At the end, the subject is categorized as “normal” (score between 70 and 100), “mild” (score between 50 and 70), or “moderate and worse” (score < 50). For “mild” and “moderate” expected hearing loss, the subject is advised to see a doctor.

#### Data and Statistical Analysis

Matlab was used for data processing, statistical analyses and for creating figures. Audiometric data and DIN test data, loaded from the text files generated by the web app, were stored in Microsoft Excel. For each subject, the status of hearing (i.e., “normal hearing” or “hearing loss”) was determined from the poorer ear PTA of each subject. Pearson correlation coefficient and linear or multilinear regressions were used to assess coevolution of different parameters like age, hearing loss and SNR score of DIN (Matlab functions: corrcoef, fit, confint, predint). Receiver operating characteristics (ROC) curves were calculated (Matlab functions: fitgml, perfcurve) to determine the sensitivity and specificity of the DIN tests for different cutoff values, to detect mild hearing loss and worse (PTA > 20 dB HL or PTA > 25 dB HL depending on the French or international standard, respectively) and moderate hearing loss and worse (PTA > 40 dB HL). Distribution of results were calculated and fitted with unimodal (one) or bimodal (two) Gaussian models (Matlab functions: hist, fitgmdist). Gaussian distribution function is $f\left(x\right)=1/\left(\sigma \surd 2\pi \right)\;e^{\wedge }\left(-{\left[\left(x-\mu \right)\right]}^{\wedge }2/\left(2\sigma^{\wedge }2\right)\right)$, with μ the mean and σ the standard deviation).

## Results

**Figure 2:**
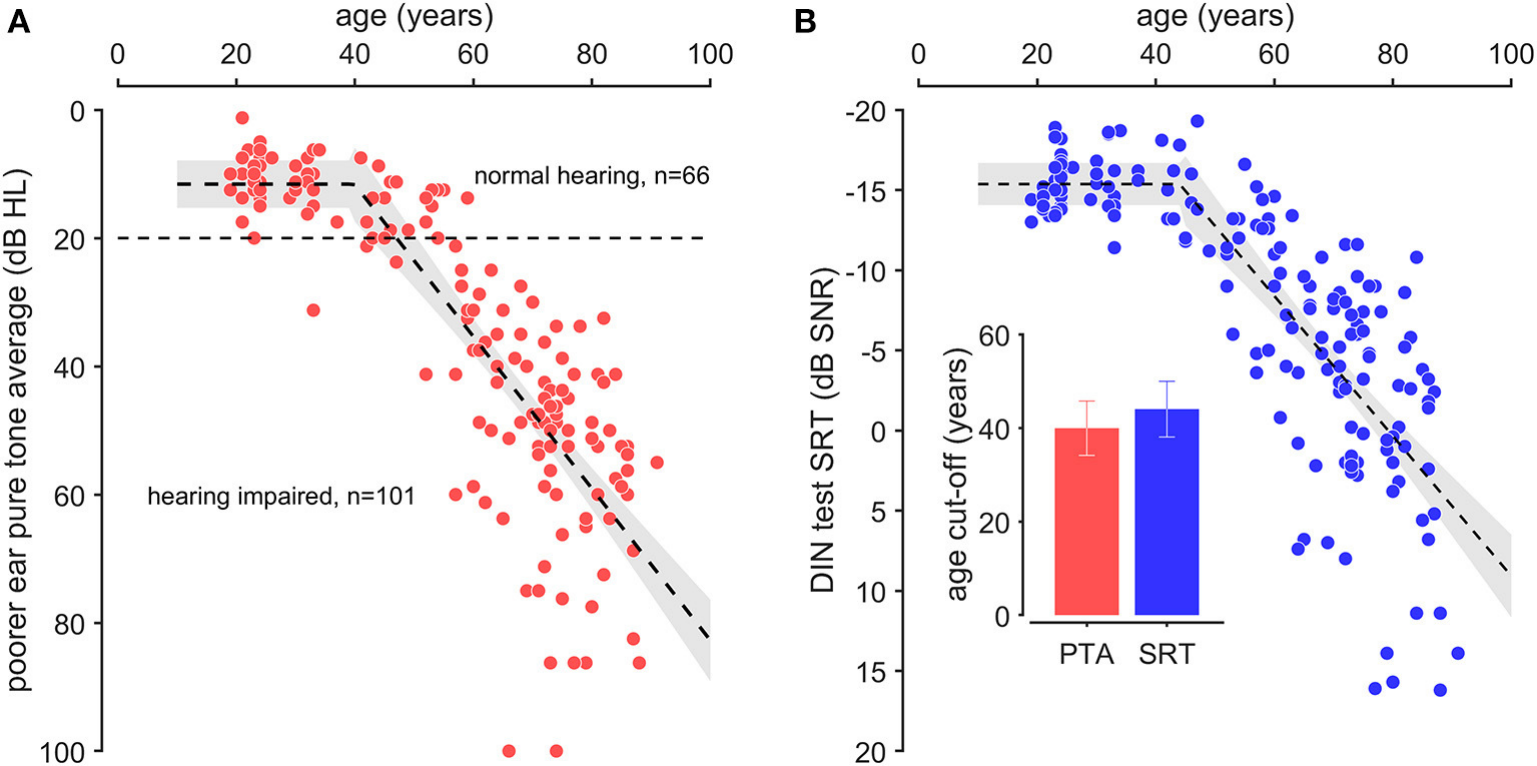
Pure tone average thresholds and digit in noise speech reception threshold as a function of age. **(A)** Pure tone average (PTA) was determined by averaging pure tone thresholds at 0.5, 1, 2, and 4 kHz from the poorer ear of each subject (red dots). **(B)** Digits-in-noise speech reception threshold (DIN SRT, blue dot). The broken-stick regression (dotted line) is presented with confidence interval (gray area). The inserted graph presents the cut-off values of broken-stick regression for PTA (red bar) and DIN SRT (blue bar). Note that the cut-off age for PTA (40 years-old) and DIN SRT (44 years-old) are not statistically different.

Pure tone average (PTA) of the poorer ear was determined by averaging the thresholds at 0.5, 1, 2, 4 kHz to obtain a single value for each ear and each participant (Figure 2A). A broken-stick model [*y* = *max (a, cAge* + *a* – *bc)*] was used to characterize the time course of PTA or SRT, in which “a” represents the normal hearing, “b” the cut-off age and “c” the slope. All parameters are given with 95% confidence interval. In our reference normative population (*n* = 167), the mean PTA was 11.6 dB HL (±3.7 dB) until a cut-off age of 40-years of age (±6 years) where the mean thresholds increase at a pace of 1.18 dB/year (±0.18 dB/year). The time course of DIN SRT was similar to PTA (Figure 2B) with a mean score of −15.4 dB SNR (±1.3 dB) up to a cut-off age of 44-years of age (±6 years) where the mean DIN SRT increase at a pace of 0.43 dB SNR/year (±0.07 dB/year). Cut-off ages of PTA and DIN SRT did not differ statistically. Despite speech perception in noise requiring more attention and auditory processing than in quiet, the similarity of PTA and DIN SRT results with ages suggest that both tests are related to peripheral hearing loss.

### Sensitivity and Specificity of the DIN Test

**Figure 3:**
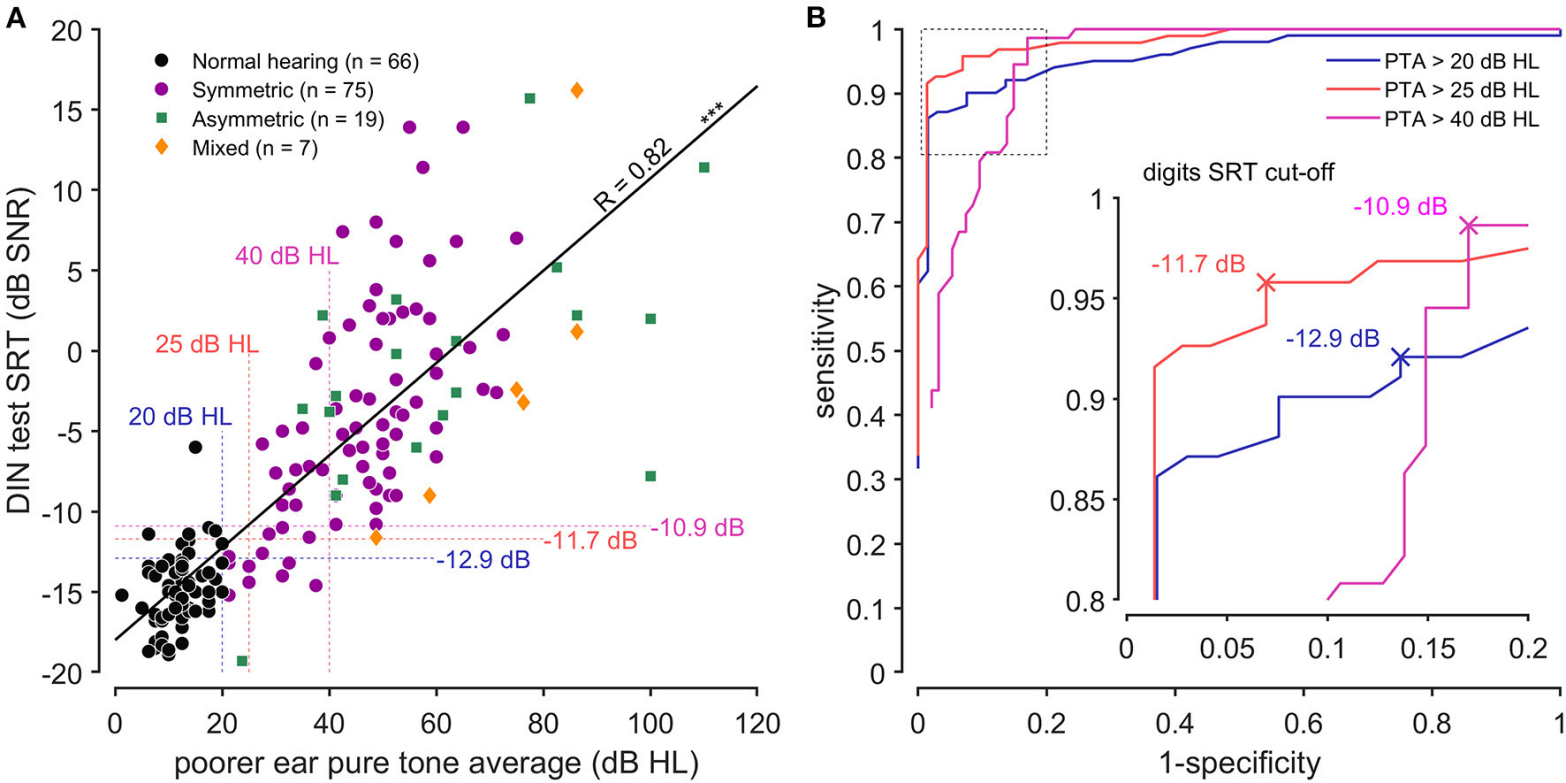
Selectivity and specificity of the DIN test. **(A)** Correlation between antiphasic digit speech reception threshold (DIN SRT) and pure tone average (PTA) from the poorer ear of each subject (*R* = 0.82, *p* < 0.001). The different symbols represent normal hearing (black dots), symmetric sensorineural loss (purple dots), unilateral or dissymmetric loss (green square) and conductive loss (orange diamonds). Vertical dotted lines represent the French standard for mild (and worse) hearing loss (PTA > 20 dB HL, blue), the international standard for mild hearing loss (PTA > 25 dB HL, red) and the moderate (and worse) hearing loss (PTA > 40 dB HL, pink). Horizontal dotted lines represent the corresponding DIN SRT cut-offs. **(B)** Shown are the ROC curves corresponding to PTA > 20 dB HL (blue line), PTA > 25 dB HL (red line) and PTA > 40 dB (pink line). The insert graph emphasizes the DIN SRT cut-offs (sensitivity and specificity).

Poorer ear PTA was significantly correlated with DIN SRT (*r* = 0.82, *p* < 0.001, Figure 3A) across all types and the degrees of hearing loss. ROC curves were calculated to determine the sensitivity (true-positive rate) and specificity (false-positive rate) of the DIN tests for different cut-off values. The optimal SNR cut-off values were chosen using a cost function that optimized the Youden index (lowest misclassification) favoring sensitivity over specificity (Figure 3B). According to French regulation, hearing is considered normal when PTA is below 20 dB (BIAP Recommendation 02.1) (40). To detect all hearing losses (PTA > 20 dB HL), a SRT cutoff value of −12.9 dB SNR corresponded to a sensitivity and a specificity of 0.92 [0.84; 0.95] and 0.86 [0.77; 0.93], respectively. For PTA > 25 dB HL, which is the international standard, a SNR cutoff of −11.7 dB corresponded to sensitivity and a specificity of 0.96 [0.9; 0.98] and 0.93 [0.84; 0.97], respectively. To detect moderate hearing loss and worse (PTA > 40 dB HL), the SNR cutoff was −10.9 dB, corresponding to a sensitivity and the specificity of 0.99 [0.92; 1] and 0.83 [0.75; 0.9], respectively.

### DIN SRT Distribution

**Figure 4:**
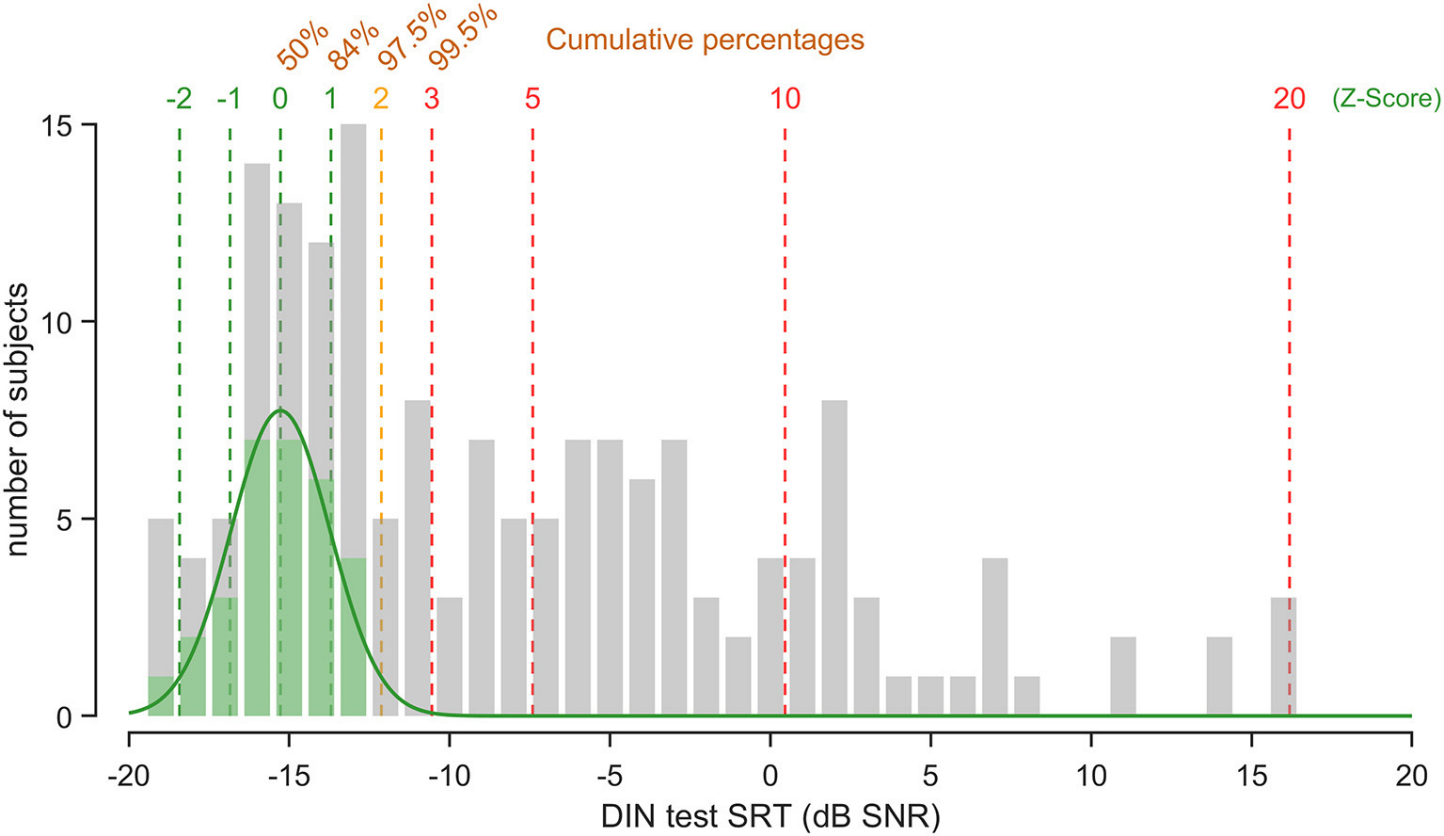
DIN SRT distribution. Shown is the DIN SRT distribution across all the subjects participating to the experiment (gray). Subjects under 25 years of age with normal-hearing (PTA < 20 dB HL) constitutes the normative population (green). Note that the DIN SRT of the normative population follows a standard normal distribution (green line, *R*^2^ = 0.97). The *Z*-scores are shown (dotted lines) with corresponding percentile values.

To establish a reference range of DIN SRT, we selected a population composed of people 25-years of age and normal PTA (*n* = 30), from which we determined the mean (−15.3 dB SNR) and standard deviation (1.6 dB) of the DIN SRT. The Z-score was calculated to associate a value of normality (Figure 4). Because the DIN SRT follows a standard normal distribution (Kolmogorov-Smirnov test, *p* < 0.001) for this subgroup of participants, a *Z*-score of 1.5 (DIN SRT ≤−12.9 dB SNR) corresponds to the 95th percentile, which is verified with the direct evaluation of percentile on data of the subgroup with exactly −13 dB SNR. In other words, a subject with a DIN SRT of −12.1 dB has a Z-score of 2 (97.5% confidence interval), and a subject with a DIN SRT of −10.5 dB has a Z-score of 3 (99.5% confidence interval).

### Mobile Höra Application

**Figure 5:**
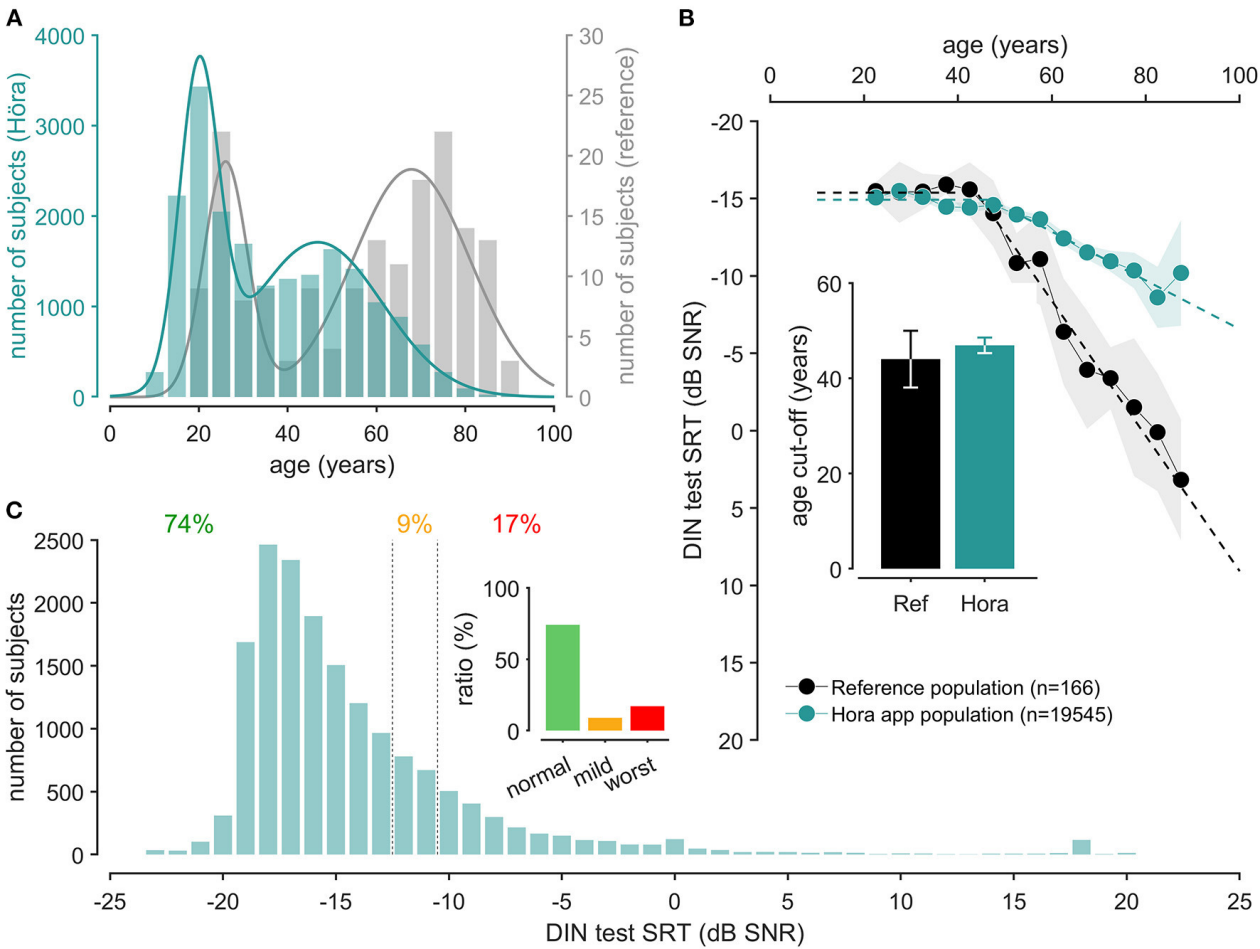
Höra screening campaign results. **(A)** Shown is the age repartition of the Höra population (green, left axis) compared to the reference population (gray, right axis). Curves correspond to the sum of two gaussian model (reference population, gray, *f*_(*x*)_ = *3.76*×*exp(–((x*−*26)/6.94)*^2^*)* + *3.66*×*exp(–((x*−*67.8)/18.5)*^2^), *R*^2^ = 0.72, Höra population, green, *f*_(*x*)_ = *668.46*×*exp(–((x*−*20)/6.24)*^2^*)* + *332*×*exp(–((x*−*78.6)/20.6)*^2^, *R*^2^ = 0.95). **(B)** Mean ± SEM of digits-in-noise speech reception thresholds for reference (gray) and Höra (green) population relative to age and the corresponding broken-stick regression. The inserted graph presents the cut-off values of broken-stick regression for reference (gray bar) and Höra (blue bar). Note that the cut-off age for PTA (44 years-old) and DIN SRT (46 years-old) are not statistically different. **(C)** Shown is the DIN SRT distribution across all the subjects participating to the Höra screening campaign (green). The inserted graph presents the distribution of Höra tests as normal hearing (74%), mild hearing loss (9%), or moderate or worst hearing loss (17%).

We subsequently integrated the French antiphasic DIN test in a mobile app (iOS and Android) called Höra. The use of the Höra smartphone app allowed us to reach a large population of subjects (*n* = 19,545 from March 26 to April 26, 2021). Overall, the proportion of male users (55.7 %) was more common than the females (45.3 %). Compared against our reference normative population, the smartphones users were younger (median age 55 and 33 years of age, respectively). Both populations showed a bimodal gaussian distribution across ages (Figure 5A). The first mode was around 26 years of age ±12 for the reference normative population versus 20 year of age ±10 for smartphones users, and the second mode was 68 year of age ±34 for the reference normative population vs. 75 year of age ±20.41 for smartphone users. For both populations, the normal SRT (−15.4 dB SNR vs. −14.9 dB SNR) and the cut-off age was similar (44 vs. 47 years). In contrast, the slope in the Höra population was less steep (0.16 dB/year ± 0.016 for smartphone users vs. 0.44 dB/year ± 0.09 for reference normative population) attesting to the proportion of hearing loss subjects being less in the Höra app users (Figure 5B). Accordingly, 74% of the subjects were classified as “normal” with DIN SRT ≤ 12.9 dB, corresponding to a PTA < 20 dBHL or a Z-score < 1.5 (Figure 5C), whereas 9% presented with a suspicion of mild hearing loss (predicted PTA between 20 and 40 dBHL or *Z*-score between 1.5 and 3) and 20 % had a moderate and worse hearing loss categorization (predicted PTA > 40 dBHL or Z-score > 3) (Figure 5C).

## Discussion

Our results support the use of the French version of the antiphasic DIN test to detect all hearing losses (symmetric and asymmetric sensorineural hearing loss and mixed hearing loss) with high sensitivity (0.92) and specificity (0.86). We further propose a standard score to compare speech in-noise results to a normal-hearing population.

The onset of the decline of DIN SRT and PTA with age was similar (44 ± 6 dB SNR and 40 ± 6 dB HL for DIN SRT and PTA, respectively). When DIN SRT was expressed as the function of the poorer ear PTA, the correlation was highly significant (*R* = 0.82, *p* < 0.001) and comparable with the English antiphasic DIN test (*R* = 0.82) (31) using the same protocol on a different population with different language. Comparing with the Jansen and colleagues study using 20 subjects (40 ears), the PTA-SRT correlation reported from our reference normative population (*n* = 167) is higher (0.82 vs. 0.77), probably because of the antiphasic presentation in our study. These results support the conclusion of Van den Borre et al. (24) suggesting that the mode of recording, the vocal material, the construction of the speech-weighted noise seems to have minor effects on the results of the DIN test, and only presentation monaural/binaural vs. antiphasic seems induce significant changes. To evaluate the impact of binaural presentation, we performed an additional study in 19 young adults (mean age 22.2 ± 3.4 years) with normal-hearing (unpublished). Using this binaural protocol, we found a mean SRT value of −10.7 ± 1.3 dB SNR, which closely match with the French test version reported by Jansen using a standard monaural DIN test (−10.2 ± 0.5 dB SNR) via a broadband headphone (42). Therefore, the more favorable DIN SRT measured in our reference population (*n* = 167, mean SNR= −15.4 ± 1.3 dB) is most likely due to the antiphasic presentation of the digits. This is consistent with the studies of Smits et al. (36) and de Sousa et al. (31, 34) in which antiphasic presentation of digits improved the average SRT with ~5–7 dB for normal hearing listeners. This improvement in SRT shift is the result of binaural masking level difference enabled using antiphasic presentation (35).

Based on the strong correlation between PTA and DIN SRT, we calculated ROC curves to determine the best DIN cut-offs predicting hearing impairment. The ROC curve depends on the correlation between the predictor and predicted values. The prediction for a mild hearing loss on the poorer ear according to the international standard (PTA > 25 dB HL) showed a sensitivity and a specificity of 0.96 and 0.93, respectively, compared to 0.95 and 0.73 for the English version (31). The test performs equally well for the prediction of moderate hearing loss (PTA > 40 dB HL) with sensitivity and specificity of 0.99 and 0.93, respectively. These values are better than the values reported for the English version (31) with 0.95 and 0.75 for sensitivity and specificity, respectively. This may be due to some differences in the representation of sub-populations like normal hearing older people or conductive loss. As highlighted by the study on English antiphasic digits in noise test (31), the test is very efficient to detect hearing losses of any type but does not give more information on their characteristics, like the degree of hearing difference for unilateral or asymmetrical losses. This would require to perform either two monaural tests like in older version of the test or a combination of binaural and antiphasic sequential testing that would increase test duration.

The French antiphasic DIN test then demonstrates very high quality of hearing loss prediction, but some outliers can be seen, i.e., false negatives and false positives, and may need some explanations. It is important to keep in mind that PTA and DIN test are subject to measurement errors that are potentialized when compared for screening. In our ROC analysis false negatives values correspond to subjects with hearing loss (PTA ≥ 20 dB HL) with a good SRT in noise (DIN SRT < −12.9 dB SNR). This phenomenon can be explained by the antiphasic stimulus presentation, known to improve speech perception in noise for people with well-preserved symmetric low frequencies hearing thresholds (35). On the contrary, false positives correspond to normal-hearing subjects with poor score in DIN test. A first explanation may be a difficulty to maintain attention through the test or a miscomprehension of the task that can occur in non-guided smartphone applications. This can also be explained by “hidden hearing loss,” defined as selective reduction of the cochlear nerve synapse number associated with noise exposure or aging (43–45) while outer hair cells remain well-preserved. In other words, false positive may present normal pure tone audiometry while being classified as hearing loss by the application. In any case, they need to be taken care of and advised to see an ENT doctor.

Next, we used the Z-score method to define statistical criteria of normality for speech in noise evaluation. The *Z*-score is very useful because it allows calculation of the probability of a score occurring within a normal-hearing population and compare two scores that come from different populations with the normal distribution. In our study, a score of 0 roughly corresponds to the normality (DIN SRT = −15.4 dB SNR). Note that DIN SRT ≤−13 dB SNR value (*Z*-score = 1.5, percentile 95) roughly corresponds to the cut-off inferred from the ROC curves (−12.9 dB SNR). Consequently, a subject with DIN SRT of −12.2 (Z-score = 2, confidence interval: 97.5%) will be ranked in the hearing loss population. A Z-score of 2 also fits with the recommendation of the French ENT society (46) (3 dB SNR above the norm) estimate from different tests of speech perception in noise (Hint, FrBio, French DIN test or Framatrix). Finally, Z-score calculation may also provide unified basis for inter-language comparison of DIN tests while SRT values ranges are different.

After implementation of the French antiphasic DIN test on iOS and Android mobile apps, it was launched as Höra. In total, 19,545 completed tests were registered and analyzed. Age distributions showed a first mode around 25 years of age and a second mode around 50 years of age. Although the smartphone users are younger than our reference normative population (median age: 33 vs. 55 years of age, respectively), the normal SNR and the cut-off age was similar in both populations, which supports the reliably of the test to predict hearing loss. Three quarters of them were classified as normal (74%) and one quarter presented mild (9%) or more severe loss (17%), which is also consistent with English version (HearZA) realized in 2018 by De Sousa et al. (47). When we compared the Höra study with the French screening test using digit triplet SRTs (42), the number of subjects who fell in the 50–70 year range was lower (23 vs. 60%, respectively) and the percentage of people with a “good” SRT outcome was higher (74 vs. 46%, respectively). The discrepancies are probably due to lower average age in the Höra population (median age: 33 vs. 58 years of age, respectively).

In summary, the present results validate the efficiency of the Höra application in its purpose of screening and raising awareness on hearing loss. Since the application counsels a confirmation by a professional, listeners with hearing loss will benefit from a follow-up diagnostic assessment by a hearing specialist who will confirm the smartphone test result. The French Ministry of Health recently outlined the full reimbursement of hearing aids (8). In addition to the implementation of this reform, screening the French population through an accessible free mobile app has significant potential to increase access to hearing aids and improve the subsequent quality of life in a larger proportion of the population.

## Data Availability Statement

The raw data supporting the conclusions of this article will be made available by the authors, without undue reservation.

## Ethics Statement

Ethical review and approval was not required for the study on human participants in accordance with the local legislation and institutional requirements. The patients/participants provided their written informed consent to participate in this study.

## Author Contributions

JCC, DS, CS, JLP, and FV designed and conceived the study. JCC and MJD coordinated the study. JCC and LG collected the data. JCC, DS, and CS performed the data and statistical analysis. JCC and JLP designed and realized the figures and wrote the first draft. All authors contributed to manuscript revision, read, and approved the submitted version.

## Funding

This work was supported by Fondation Pour l'Audition, non-profit foundation recognized as a public-interest since 2015.

## Conflict of Interest

The authors declare that the research was conducted in the absence of any commercial or financial relationships that could be construed as a potential conflict of interest.

## Publisher's Note

All claims expressed in this article are solely those of the authors and do not necessarily represent those of their affiliated organizations, or those of the publisher, the editors and the reviewers. Any product that may be evaluated in this article, or claim that may be made by its manufacturer, is not guaranteed or endorsed by the publisher.
